# Severe Hypoglycemia in a Juvenile Diabetic Rat Model: Presence and Severity of Seizures Are Associated with Mortality

**DOI:** 10.1371/journal.pone.0083168

**Published:** 2013-12-30

**Authors:** Margaret Maheandiran, Shanthini Mylvaganam, Chiping Wu, Youssef El-Hayek, Sonia Sugumar, Lili Hazrati, Martin del Campo, Adria Giacca, Liang Zhang, Peter L. Carlen

**Affiliations:** 1 Toronto Western Research Institute, University Health Network, Toronto, Ontario, Canada; 2 Department of Physiology, University of Toronto, Toronto, Ontario, Canada; 3 Tanz Centre for Research in Neurodegenerative Diseases, University of Toronto, Toronto, Ontario Canada; 4 Department of Neurology, Toronto Western Hospital, Toronto, Ontario, Canada; Max-Delbrück Center for Molecular Medicine (MDC), Germany

## Abstract

It is well accepted that insulin-induced hypoglycemia can result in seizures. However, the effects of the seizures, as well as possible treatment strategies, have yet to be elucidated, particularly in juvenile or insulin-dependent diabetes mellitus (IDDM). Here we establish a model of diabetes in young rats, to examine the consequences of severe hypoglycemia in this age group; particularly seizures and mortality. Diabetes was induced in post-weaned 22-day-old Sprague-Dawley rats by streptozotocin (STZ) administered intraperitoneally (IP). Insulin IP (15 U/kg), in rats fasted (14–16 hours), induced hypoglycemia, defined as <3.5 mM blood glucose (BG), in 68% of diabetic (STZ) and 86% of control rats (CON). Seizures occurred in 86% of STZ and all CON rats that reached hypoglycemic levels with mortality only occurring post-seizure. The fasting BG levels were significantly higher in STZ (12.4±1.3 mM) than in CON rodents (6.3±0.3 mM), resulting in earlier onset of hypoglycemia and seizures in the CON group. However, the BG at seizure onset was statistically similar between STZ (1.8±0.2 mM) and CON animals (1.6±0.1 mM) as well as between those that survived (S+S) and those that died (S+M) post-seizure. Despite this, the S+M group underwent a significantly greater number of seizure events than the S+S group. 25% glucose administered at seizure onset and repeated with recurrent seizures was not sufficient to mitigate these continued convulsions. Combining glucose with diazepam and phenytoin significantly decreased post-treatment seizures, but not mortality. Intracranial electroencephalograms (EEGs) were recorded in 10 CON and 9 STZ animals. Predictive EEG changes were not observed in these animals that underwent seizures. Fluorojade staining revealed damaged cells in non-seizing STZ animals and in STZ and CON animals post-seizure. In summary, this model of hypoglycemia and seizures in juvenile diabetic rats provides a paradigm for further study of underlying mechanisms. Our data demonstrate that severe hypoglycemia (<2.0 mM) is a necessary precondition for seizures, and the increased frequency of these seizures is associated with mortality.

## Introduction

The brain stores minimal glucose, mainly in the form of astrocytic glycogen [1]–[3] and is dependent upon a regular supply of glucose from circulating blood [4]. Hypoglycemia is the major limiting factor in the management of Type 1 diabetes [5]–[8] with patients experiencing an average of 2 episodes per week [9]. More severely, hypoglycemia can lead to seizures and coma, with generalized seizures being the major acute complication [10] occurring in children [11] and adolescents [12]. In addition, devastating effects, such as the “dead in bed” syndrome, possibly due to hypoglycemic seizures [13], occur approximately 3 times more frequently in young people with diabetes than in those without [14].

Studies evaluating the relationship between hypoglycemia and cognitive dysfunction have yielded conflicting results. Some studies have suggested that hypoglycemia results in cognitive decline [15]–[17] whereas others have demonstrated that cognition was not impaired long-term [18]–[20]. Perantie et al [21] showed a reduction particularly in spatial learning scores only after repeated hypoglycemic episodes with the initial episode occuring before the age of 5. A potential reason for this disparity in the literature, is that seizure incidence was not considered a variable when assessing the effects on cognition. Yet, seizures can exacerbate the effects of hypoglycemia-induced dysfuntion in humans [22] and in an animal model [23]. Very severe hypoglycemia (<1.0 mM) can result in neuronal injury [24]–[26] with seizures further exacerbating these effects [26]. Furthermore, recurrent hypoglycemic episodes diminish the premonitory symptoms of hypoglycemia (hypoglycemia unawareness) and the counterregulatory response to subsequent hypoglycemia (hypoglycemia-associated autonomic failure), thus jeopardizing patient safety [5], [8], [9], [27], [28].

Critically, both hyperglycemia and hypoglycemia can upset the balance between inhibition and excitation of neuronal networks and have been demonstrated to increase seizure susceptibility. A high-glucose medium is associated with reduced seizure threshold in vitro [29] and glucose deprivation can increase neuronal excitability [30], [31]. Abdelmalik et al [23] demonstrated that glucose deprivation alone can produce ictal activity in the hippocampus of a juvenile mouse and subsequently exacerbate synaptic dysfunction. The loss of high-energy substrates, such as glucose, leads to the release of excitatory amino acids such as glutamate that promote hyperexcitability and consequent excitotoxicity [3]. In addition, K_ATP_ channel expression decreases in overnight fasted rodents, therefore contributing to neuronal excitability and possibly to hypoglycemic seizures [32]. At basal expression levels, these channels play a neuroprotective role by hyperpolarizing neurons during energy shortages thus mitigating expenditure on action potential firing [31]. Previous animal studies of severe hypoglycemia (blood glucose < 1.0 mM) have shown that neuronal damage initially occurs in the cerebral cortex as well as in the CA1 and dentate gyrus regions of the hippocampus [26], [33] and subsequently in the basal ganglia and thalamus. Neurons in the brain stem, cerebellum, and spinal cord are generally spared, as are glial cells and white matter tracts. This pattern is also observed in human autopsy studies [33].

In the developing brain, excitation is predominant [34] and the increased propensity for seizures or seizure-like activity has been shown in several experimental models [35]. Age-related differences in seizure susceptibility may be due to functional differences in the substantia nigra pars reticulata (SNR). In adult rats, the SNR is functionally separated into the anterior anticonvulsant and posterior proconvulsant regions. However in rats, aged 15–21days, only the proconvulsant region is present. [36].

EEG abnormalities are more frequent in patients with IDDM than in the general population [37], [38] and are associated with hypoglycemia [39]. However, a clinical study revealed no clear EEG abnormalities during tonic-clonic seizures [40]. Similarly, severe seizure-like behaviours were not associated with electrographic abnormalities recorded in the cerebral cortex and hippocampus of nondiabetic adult rats [41]. While, subcortical structures such as the SNR are involved in the control of the motor component of hypoglycemic seizures [32], [42], the origin of these seizures remains unknown. Though other studies have used STZ to induce diabetes in young animals [43], [44], to our knowledge, hypoglycemic seizures have not been assessed in this age group; a population most susceptible to such events. Therefore, we have established an STZ model for Type 1 diabetes in juvenile rats to describe hypoglycemic seizures, establish seizure thresholds, evaluate the efficacy of anticonvulsants, and measure histological and electrophysiological correlates.

## Materials and Methods

### Animals

All studies were done in accordance with and approved by the Animal Research Council at the University Health Network (Toronto, Ontario, Canada). Male Sprague Dawley rats from Charles River Laboratories (21-day-old, weaned), weighing 40–60 g, were housed in pairs in a temperature-controlled environment with *ad libitum* access to water, a standard rat chow diet and under a 12-hour light/dark cycle. The rats were ear-punched for identification.

### Induction of Diabetes

Streptozotocin (STZ) was dissolved in a citric acid buffer (0.1 mM sodium citrate (Fisher Scientific) buffered with 1 M citric acid (Fisher Scientific) to a pH of 4.5) making a 10 mg/ml solution just prior to injection. The 22-day-old rats were fasted overnight (14–16 hrs) and received IP injections with one of the following doses of STZ: 60 mg/kg, 75 mg/kg or 80 mg/kg, to induce diabetes. Controls (CON) were randomly selected and injected with the 0.1 mM sodium citrate buffer vehicle. Following STZ or citric buffer vehicle injections, body weights and tail vein blood glucose levels were measured (BG) using a Hemocue Glucose 201 glucometer (Hemocue, Vitaid). Measurements were made 2 days after STZ IP and every 4 days subsequently to confirm stable diabetes.

### Induction of Hypoglycemia

Animals were fasted overnight (14–16 hours), administered insulin IP (15 units/kg; Humulin R; Eli Lilly and Company) and video-monitored for 5 hours to detect motor seizures or convulsive seizure-like events, these are referred to as “seizures” for the rest of this paper. Fasting BG was measured (see methods above) prior to insulin IP and hourly, after receiving insulin, leading up to seizures. The BG measures were conducted hourly, as the glucose meter (Hemocue) used was more accurate but required substantially more blood volume than over-the-counter glucose meters. “Lowest BG” was considered as the minimum blood sugar measured in the time period leading up to seizures or up to 3 hours in animals that did not seize. In some cases this measure was taken 15–30 mins prior, as blood samples were difficult to acquire when rats were lethargic. In a subset of animals (22 diabetic and 16 control rats), BG was measured at seizure onset before administering treatment to determine the BG threshold for seizures. After seizures, rats that appeared healthy as defined by resumption of normal grooming habits as well as eating and drinking, survived. Rats that continued seizing despite treatment, were not responsive and displayed agonal breathing (Table S1) were euthanized and brains were collected for histological analysis. These animals were categorized as non-surviving with seizures and mortality (S+M) in all further analyses.

### Treatment of Seizures

*The strategies that were employed to treat seizures are described in **Table 1**. CON rats were used in the **glu** treatment group and STZ rats were used in all three of the treatment groups.*

**Table 1:** Treatment strategies that were employed to treat or attenuate seizures.

Treatment	GLU	AC+1XGLU	C AC+MULTIPLE GLU
At Seizure Onset	1 g/kg 25% glucose in saline	Diazepam 5 mg/kg+Phenytoin 50 mg/kg+25% glucose saline	Diazepam 5 mg/kg+Phenytoin 50 mg/kg+25% glucose saline
Subsequent Seizures	0.5 g/kg glucose	Diazepam 2.5 mg/kg	0.5 g/kg glucose for seizures or BG<2.5 mM

### Seizure Scoring

A seizure score was developed to characterize and quantify the SLEs observed in this model (**Table 2**). The behaviours in this score chart were based on previous scores [45] and modified according to our observations. Treatment was administered to rodents who displayed obvious twitches (seizure score ≥ 2.5).

**Table 2:** Seizure score to characterize and quantify the observed seizures.

Score	Behaviour
0.5	Head up or tail stiff
1	Rearing and Falling
1.5	Myoclonic jerk or hindlimbs/forelimbs stretched
2.0	Body extended with hindlimb or forelimb digging or body curled
2.5	Unilateral forelimb or hindlimb clonus
3	Ipsilateral or contralateral forelimb and hindlimb clonus
3.5	Bilateral forelimb or hindlimb clonus
4	Wild running
4.5	All limbs clonus
5	Head bent backwards forelimb clonus with tonus of hindlimbs
5.5	Partial barrel roll
6	Full barrell roll
7	Full tonic extension

The seizure score that was obtained in 5-minute epochs was used for further analysis. Animals were subsequently grouped according to the types of seizures that they underwent. Two types of categorization were performed. Firstly, rats were segregated by whether or not the brainstem was recruited (defined by the loss of righting reflex) during the seizure (seizure score ≥ 4.0). Secondly, rats were also placed in three categories (1) partial seizures: only involving one limb (seizure score  =  2.5); (2) partial to secondary generalization: the seizure spreads from one limb to other areas (3) generalization without prior evident partial seizures (seizure score ≥ 3.0).

### Statistical Analyses

All statistical tests were performed with Sigma Stat software (11^th^ version; Systat Software Inc.). Comparison of data between two groups was carried out via Students’ T-test. To compare proportions of the various treatment groups, a Chi-squared (n ≥ 5) or Fisher’s exact test (n < 5) was used. Significance was set at P<0.05. All error bars indicate ± SEM.

### EEG Monitoring

17 STZ and 12 CON rats were implanted with intracranial electrodes using the methodology previously described [41]. Data acquisition and analysis were conducted using pCLAMP 9.0 (Molecular Devices, Inc., California U.S.). 2 STZ animals had electrodes in the left motor cortex and right hippocampal formation (CA1) and the remaining 15 STZ and 12 CON rats were implanted in the right hippocampal (CA1) and left mesencephalic reticular formation. Animals were allowed 5-7 days recovery prior to hypoglycemic insult and EEG recording. Continuous EEG recordings were obtained one hour prior to insulin administration to establish a baseline (after overnight fast). Rodents were then recorded for up to 5 hours after insulin IP or until seizures occured. Simultaneous video monitoring was performed on all animals.

### Histology

48 hours after the hypoglycemic episode, 7 STZ and 5 CON rats, randomly selected, were transcardially perfused with 100 mL 0.1% Phosphate Buffered Saline (PBS) and 40 mL 4% paraformaldehyde. The brain was removed at either 48 hours post-hypoglycemic insult or in age-matched STZ and CON animals. Whole brains were cut along the coronal plane into 3 mm sections and embedded in paraffin. Subsequently, paraffin blocks were cut into coronal slices, 10 microns in thickness every 50 microns with a total of 3 slices taken from each block. Fluorojade C (emission: 450 nm, excitation 530 nm) staining was performed to mark degenerating neurons. Staining methods were in accordance with the manufacturer’s protocol (millipore.com) and cells that displayed fluorescence were counted as having undergone neurodegeneration.

## Results

### Optimization of STZ dosage in Juvenile Rodents

While the STZ model of diabetes induction has been widely demonstrated in adult rodents [46] and more recently in younger animals [43], [44], a model of hypoglycemic seizures in young diabetic animals has not been established. Though 40–60 mg/kg of STZ has been sufficient to induce diabetes in adult animals [47], younger animals require a more potent dose as their pancreatic β-cells are still developing. As such, dose optimization experiments were performed. Diabetes was confirmed through blood glucose (BG) measurement from a tail vein blood sample after STZ administration. Hyperglycemia (BG > 11.1 mM) was used as the inclusion criterion for diabetes. The dosage of 60 mg/kg failed to induce diabetes in any animal (n = 16). A 50% success rate was observed with the 75 mg/kg dose (n = 28). 80 mg/kg of STZ was 91% (n = 44) successful in inducing stable diabetes in rats, and was selected for future experiments.

A week after STZ administration, the diabetic (STZ) animals had a significantly decreased weight gain (p<0.005) body weight; 78.7±1.2 g (n = 40) than the age-matched control (CON) animals; 100.5±2.5 g, (n = 17).

### Incidence of Hypoglycemia, Seizures and Mortality

To assess the prevalence of seizures and other concomitant effects of hypoglycemia, insulin IP (15 U/kg), was administered to overnight-fasted rats 1 week after STZ (diabetic rats, N = 63) or CON (controls, N = 23) induction. Overnight fasting, prior to receiving insulin, has been demonstrated to increase seizure incidence [32]. After insulin administration, the rats were video-monitored for 4-5 hours and hypoglycemia was confirmed through BG levels measured from the tail vein.

**Figure 1:**
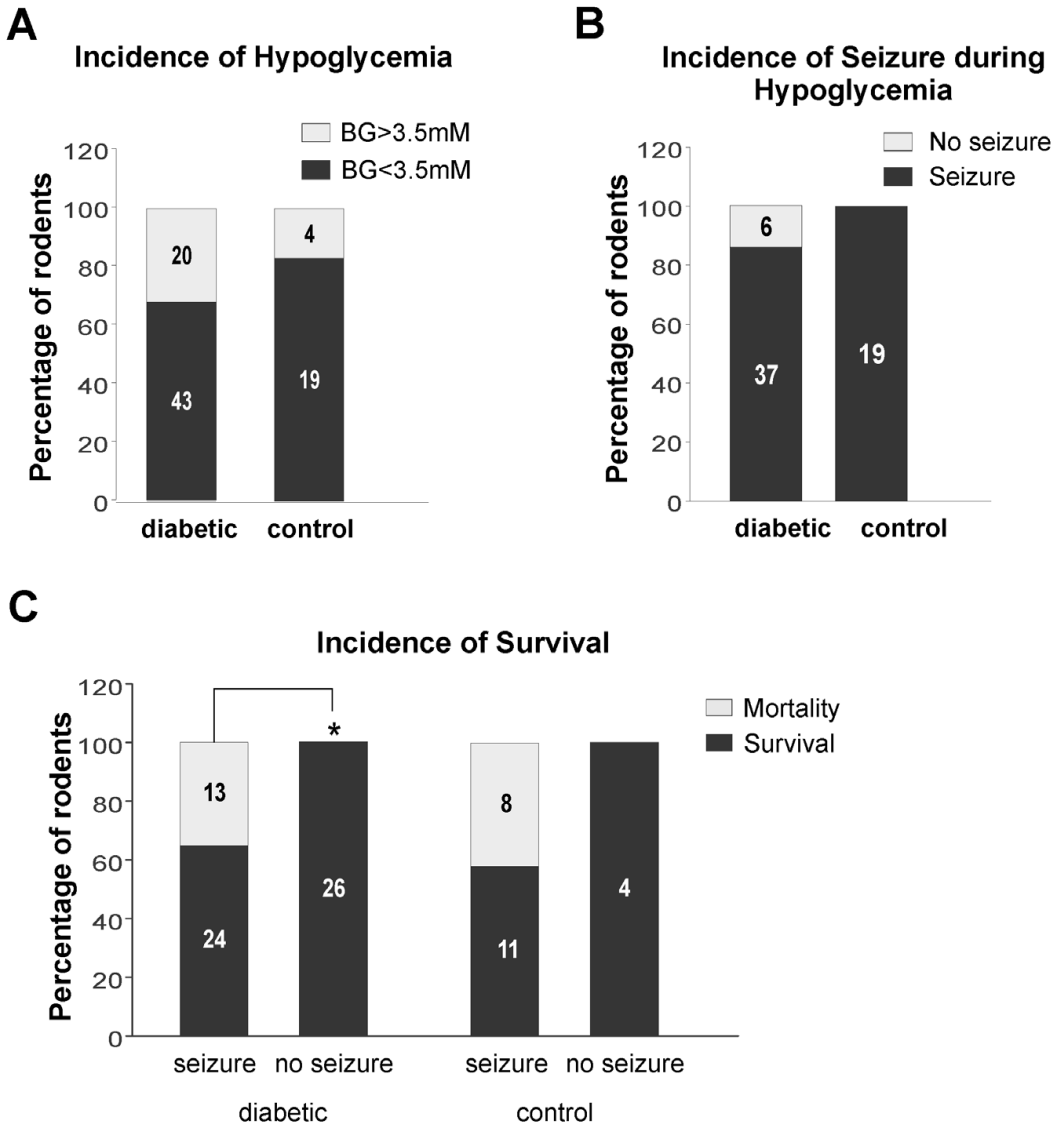
Incidence of hypoglycemia, seizures and mortality after insulin IP (15 u/kg) in overnight-fasted PN 28-30 rats; 1 week after STZ (diabetic rats) or CON (controls). **A**: Incidence of hypoglycemia in all animals injected with insulin; 68% of STZ (n = 43/63) and 83% of CON (n = 19/23); no statistically significant difference between both groups **B**: Incidence of seizures in rodents where hypoglycemia was confirmed (BG<3.5 mM); 86% of STZ (n = 37/43) and 100% of CON (n = 19/19); no significant difference exists between the two groups **C**: Rate of mortality after insulin administration and hypoglycemia; 35% (n = 13/37) of STZ and 42% (n = 8/19) of CON rats. 0% mortality observed in non-seizing STZ (n = 0/26) and CON (n = 0/4) rodents. (*) Denotes a statistically significant difference in survival between seizing and non-seizing rats in the STZ group (p<0.005).

Both CON and STZ rats, attained hypoglycemia (BG < 3.5 mM). Incidence of hypoglycemia was 68% (n = 43/63) in STZ and 86% (n = 19/23) in CON rats (**Figure 1A** **)**, which is a non-significant difference. In addition, there was no significant difference between the proportions of hypoglycemic CON (100%: n = 19/19) and STZ (86%: n = 37/43) rats that displayed convulsions (**Figure 1B**). Only 6 of the 26 non-seizing STZ rats and none of the non-seizing CON rats reached hypoglycemic levels. Mortality resulted in 35% (n = 13/37) of STZ and 42% (n = 8/19) of CON rats that exhibited seizures. Conversely, all non-seizing STZ (n = 26: p<0.005) and CON (n = 4) animals survived (**Figure 1C** **).**

### Blood Glucose Decrease within the First Hour is Predictive of Seizure but not Mortality

**Figure 2:**
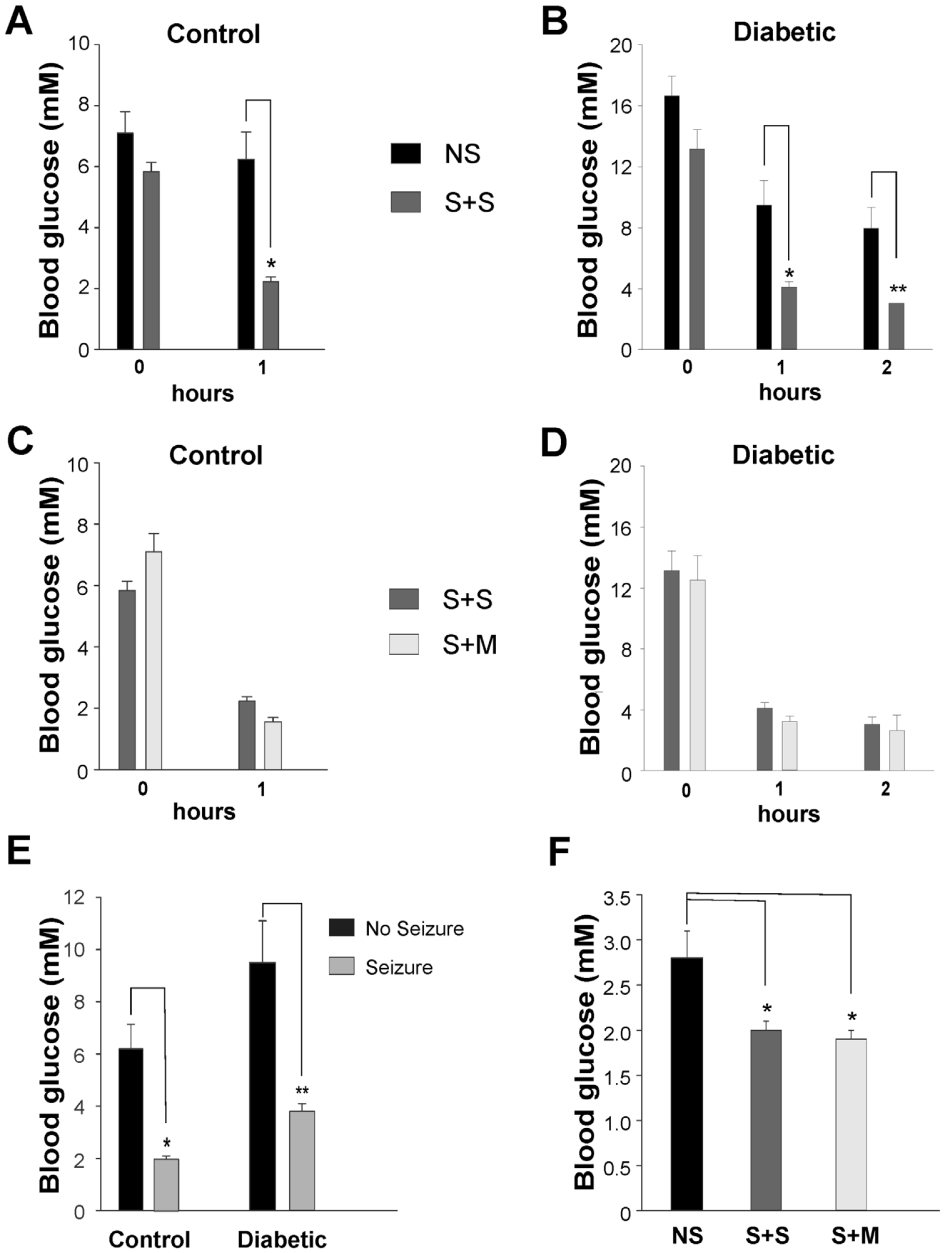
Relationship of blood glucose (BG) decrease after insulin IP (15 u/kg) to seizure and survival. **A:** No significant difference in BG of CON rats with or without seizures at 0hr (time of insulin administration). At 1 hr, BG in non-seizure (NS) group was significantly higher (*) than the seizure + survival (S+S); (p < 0.001) **B:** No significant difference in BG of STZ rats with or without seizures at 0hr (time of insulin administration). BG in NS group was significantly higher, at 1 hr (*) and 2hr (**) post-insulin, than the S+S group; (p < 0.001) **C:** No significant difference in BG of CON rats at 0hr or 1hr post-insulin in S+S and S+M groups **D:** No significant difference in BG of STZ rats at 0hr, 1hr or 2hr post-insulin in S+S and S+M groups **E:** BG levels at 1 hr post-insulin is significantly greater in CON; NS: 6.2±0.9 mM than CON with seizure: 2.0±0.1 mM (*), and in STZ; NS: 9.5±1.6 mM compared with STZ with seizure: 3.8±0.3 mM (**); (p<0.001) **F:** Lowest BG measured is significantly higher in the NS group (n = 6): 2.8±0.3 mM compared with either S+S (n = 24): 2.0±0.1 mM or S+M (n = 13): 1.9±0.1 mM groups (*); (p<0.002).

Blood glucose (BG) levels were measured prior to insulin administration and every subsequent hour until seizure onset. Fasting BG levels, prior to insulin administration, were significantly higher in STZ than in CON animals (p<0.001). Consequently, the latency to seizures was significantly increased in STZ (2.4±0.2 hours) compared to CON rats (1.1±0.1 hours, p<0.001). However, fasting BG levels were not a significant factor in CON (**Figure 2A** **)** or STZ rats (**Figure 2B** **;** **Table 3** **),** for predicting the incidence of seizures. Concurrently, prevalence of mortality was not significantly affected by fasting pre-insulin BG levels in CON (**Figure 2C**) or STZ rats (**Figure 2D** **).**

**Table 3:** Mean blood glucose level measured hourly related to outcome; (±S.E.M).

	N	0 hr	N	1 hr	N	2hr
NS	27	16.7±1.3	18	9.5±1.6	21	8.0±1.4
S+M	16	12.5±1.7	10	3.2±0.4	6	2.6±1.0
S+S	27	13.2±1.3	26	4.1±0.4	17	3.1±0.5

NS: No Seizure.

S+M: Seizure + Mortality.

S+S: Seizure + Survival.

After insulin administration, both STZ and CON rats that eventually developed seizures had a significant drop in BG levels within the first hour compared to the NS groups (1hr BG in CON: NS  =  6.2±0.9 mM, CON: seizure  =  2.0±0.1 mM, STZ: NS  =  9.5±1.6 mM, STZ: seizure  =  3.8±0.3 mM, **Figure 2E** **,** p<0.001 for both comparisons).

In the STZ group, the 37 rats displaying seizures (lowest BG  =  2.0±0.1 mM), reached significantly lower (p<0.002) BG levels than the 6 NS hypoglycemic animals (lowest BG  =  2.8±0.3 mM). There was no significant difference in the lowest BG measured between seizing rats that survived (S+S  =  2.0±0.1 mM) or died (S+M  =  1.9±0.1 mM, **Figure 2F** **;** **Table 3**). Control NS rodents did not achieve hypoglycemic levels.

### Blood Glucose Threshold for Seizure

**Figure 3:**
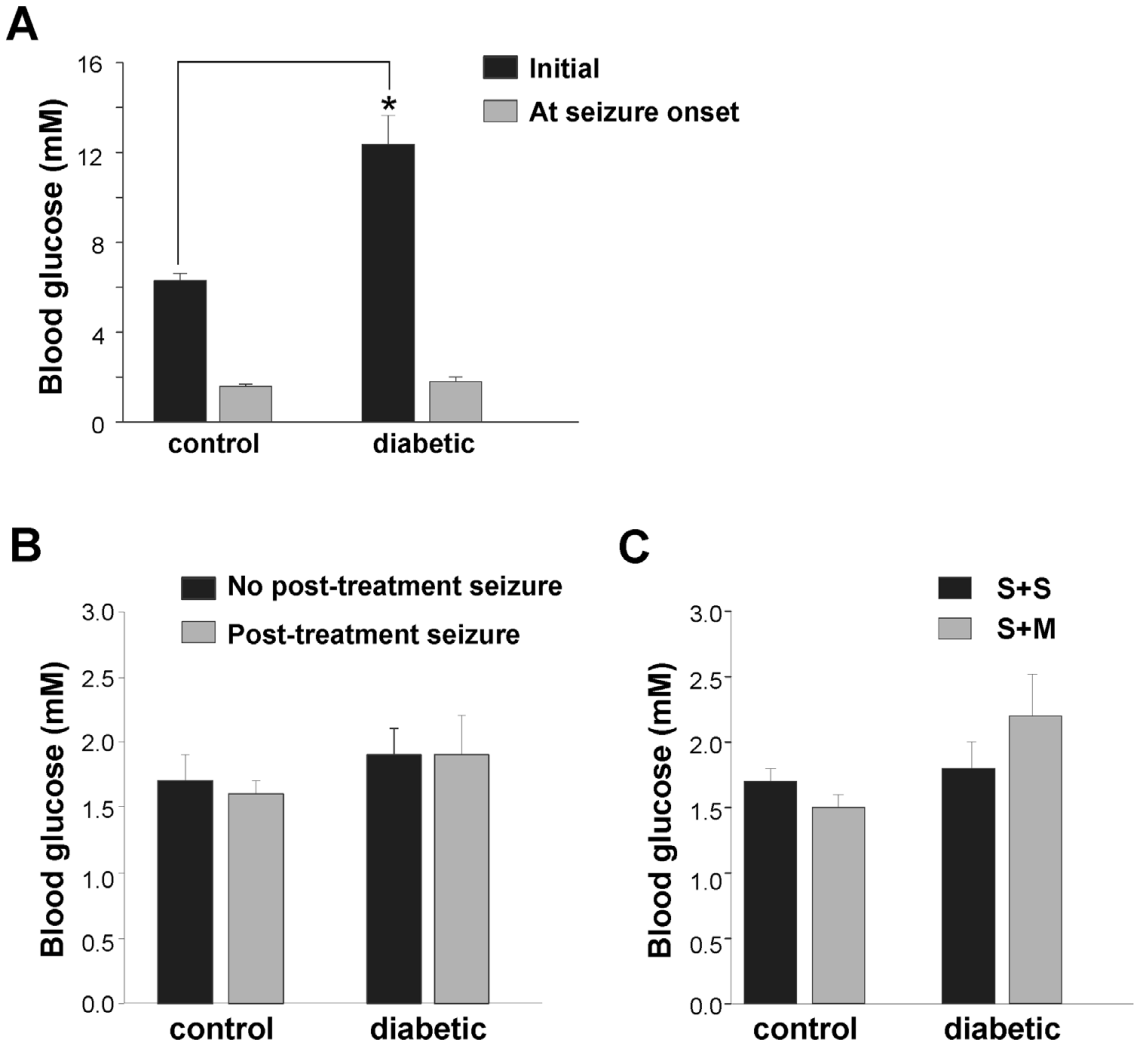
Blood glucose (BG) threshold for seizures in CON vs. STZ rats and association with survival and seizures after treatment. **A**. Initial BG (prior to insulin IP) is significantly lower in CON (6.3±0.3 mM; n = 16) than in STZ rats (12.4±1.3 mM; n = 22) (p<0.01). BG at seizure onset (observed seizure score ≥2.5) is similar in CON (1.6±0.1 mM) and STZ rats (1.8±0.2 mM) **B**: No significant association between BG levels at seizure onset and post-treatment seizures. BG levels glucose at seizure onset for CON: 1 seizure; 1.7 ±0.2 mM (n = 11) and >1 seizure; 1.6±0.1 mM (n = 5) and STZ: 1 seizure; 1.9±0.2 mM (n = 10), >1 seizure; 1.9±0.3 mM (n = 12) are not statistically different C: No significant difference between BG levels at seizure onset comparing survival and mortality. BG for CON: S+S; 1.7±0.1 mM (n = 11) and S+M; 1.5 ±0.1 mM (n = 5); STZ: S+S; 1.8±0.2 mM (n = 17) and S+M; 2.2±0.32 mM (n = 5) are not significantly different.

To establish the BG level for seizure threshold, BG at seizure onset (defined as a seizure score ≥2.5, refer to **Table 1**) was measured in 22 STZ and 16 CON rats. As mentioned above, prior to insulin administration, BG levels in STZ (12.4±1.3 mM) were significantly higher than in CON rats (6.3±0.3 mM; p<0.01) (**Figure 3A** **)**. Despite this, at the onset of seizures, the BG levels were similar in STZ (1.8±0.2 mM) and CON rats (1.6±0.1 mM; **Figure 3A**) due to the comparable decrease in the mean rates of BG in STZ (4.4±0.36 mM/hr) and CON rats (4.5±0.5 mM/hr) as well as the significantly higher latency to seizures in STZ (2.4±0.2 hours) than in CON (1.1±0.1 hours) rats (p<0.001).

BG at seizure onset was not predictive of whether rats would undergo a single seizure (mitigated by glucose administration) or subsequent seizures (despite treatment). In CON rats, the BG levels were 1.7±0.2 mM (n = 11) and 1.6±0.1 mM (n = 5) in groups with single and multiple seizures, respectively. Likewise, in STZ rats, BG levels at seizure onset of rats with a single seizure and multiple seizures were 1.9±0.2 mM (n = 10) and 1.9±0.3 mM (n = 12), respectively (**Figure 3B**). Although the seizures occurred within a limited hypoglycemic range, the BG during recovery, measured hourly post-seizure, was variable (**Figure S1 A-B**). No significant difference was measured in the seizure onset BG levels of STZ surviving (1.8±0.2 mM, n = 17) and non-surviving rats (2.2±0.3 mM, n = 5). The BG of CON rats did not differ significantly between those that survived (BG 1.7±0.1 mM, n = 11) and those that did not (BG 1.5±0.1 mM, n = 5; **Figure 3C**).

### Continued Seizures are associated with mortality

In order to establish a seizure model, the observed seizure behaviours must first be described. Reliable video recordings were obtained in 22 STZ and 16 CON rats that displayed seizures. All rats were treated with glucose at seizure onset. Seizure scoring was performed to quantify the severity of the observed seizures (**Table 2** **)**. Prior to reaching seizure-inducing hypoglycemic levels, all rats that reached moderate hypoglycemia (<3.5 mM) became lethargic. In the absence of EEG recording, it was difficult to discern less severe seizure-like activity (seizure score: 0.5–2) from lethargic behavior. Therefore, only rats that reached a seizure score of ≥2.5 (single limb clonus) were treated and classified as having seized (see methods). Seizures in the STZ and CON animals exhibited variable progression, whether or not the animals survived (**Figure S2 A-D**). Notably, mortality occurred in all rats that reached a seizure score of 7.

**Figure 4:**
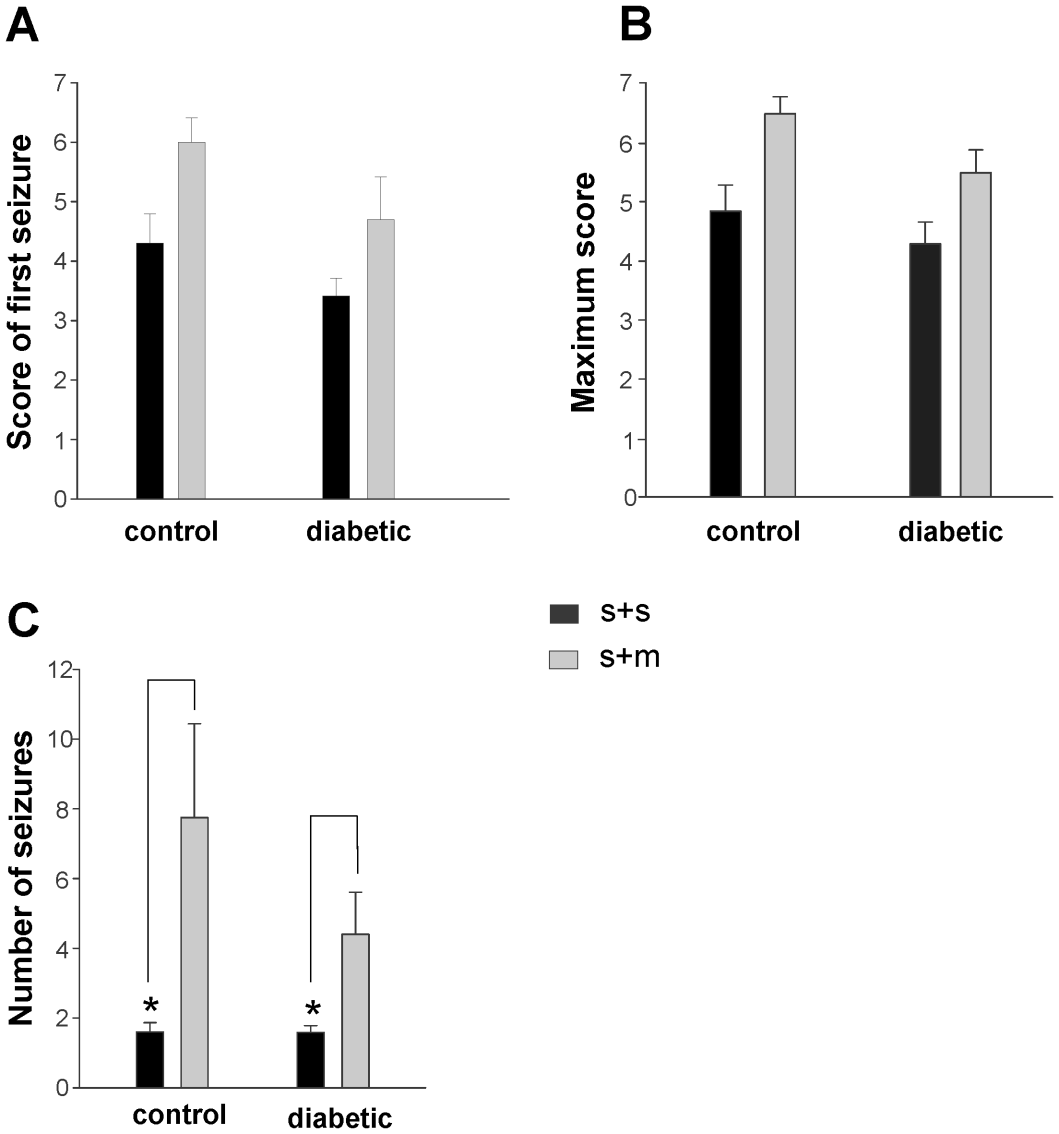
Comparing the association between seizure severity and mortality in CON and STZ rats. **A:** No significant difference in the mean score of the first seizure treated in CON rats: S+S: 4.3±0.5 (n = 11) and S+M: 6.0±0.4 (n = 5) and STZ rats: S+S: 3.4±0.3 (n = 17) and S+M: 4.7±0.7 (n = 5) **B:** No significant difference in the mean maximum seizure score observed in CON rats: S+S: 4.9±0.4 (n = 11) and S+M: 6.5±0.3 and STZ rats: S+S: 4.3±0.4 (n = 17) and S+M: 5.5±0.4 (n = 5) **C:** A statistically significant difference in the mean number of seizures between (*) CON rats: S+S: 1.6±0.3 (n = 11) and S+M: 7.8±2.7 (n = 5; p<0.01) and between (*) STZ rats: S+S: 1.6±0.2 (n = 17) and S+M: 4.4±1.2 (n = 5; p<0.001).

While it is evident that hypoglycemia and the resulting seizures are associated with mortality, it is not clear whether seizures were the sole cause (**Figure 3C**). Therefore, the analysis of seizure scores was used to isolate the effects of seizures on mortality. The severity of the first seizure, prior to treatment with glucose, was not significantly different in CON rats: S+S  =  4.3±0.5 (n = 11) and S+M  =  6.0±0.4 (n = 5) as well as STZ rats: S+S: 3.4±0.3 (n = 17) and S+M: 4.7±0.7 (n = 5) (**Figure 4A**). The most severe seizure, measured by the maximum seizure score, experienced by CON rats was statistically similar in S+S  =  4.9±0.4 (n = 11) and S+M rats  =  6.5±0.3 (n = 5, p>0.05), as was the case for the STZ rats: S+S  =  4.3±0.4 (n = 17) and S+M  =  5.5±0.4 (n = 5, p>0.05) (**Figure 4B**). Frequent BG measures could not be obtained to determine the duration of hypoglycemia. Instead, the period in which seizures (scores ranging from 0.5–7) occurred was used as an indication of hypoglycemia. These data were also statistically similar between S+M and S+S rats, whether or not they had diabetes (**Figure S4**). The S+M rats in both the STZ and CON groups trended toward a higher initial and maximum seizure score compared to the respective S+S rats. The lack of significance is potentially due to the low sample size of the S+M animals.

A stronger predictor of mortality was the number of seizures that an animal underwent. The number of seizures observed was significantly higher in the CON rats: S+M  =  7.8±2.7 (n = 5) compared with S+S  =  1.6±0.3 (n = 11; p<0.01). This difference was also observed between STZ rats: S+S  =  1.6±0.2 (n = 17) and S+M  =  4.4±1.2 (n = 5); p<0.001 (**Figure 4C** **).**

### Anticonvulsants Reduce Seizure Incidence but Not Mortality

**Figure 5:**
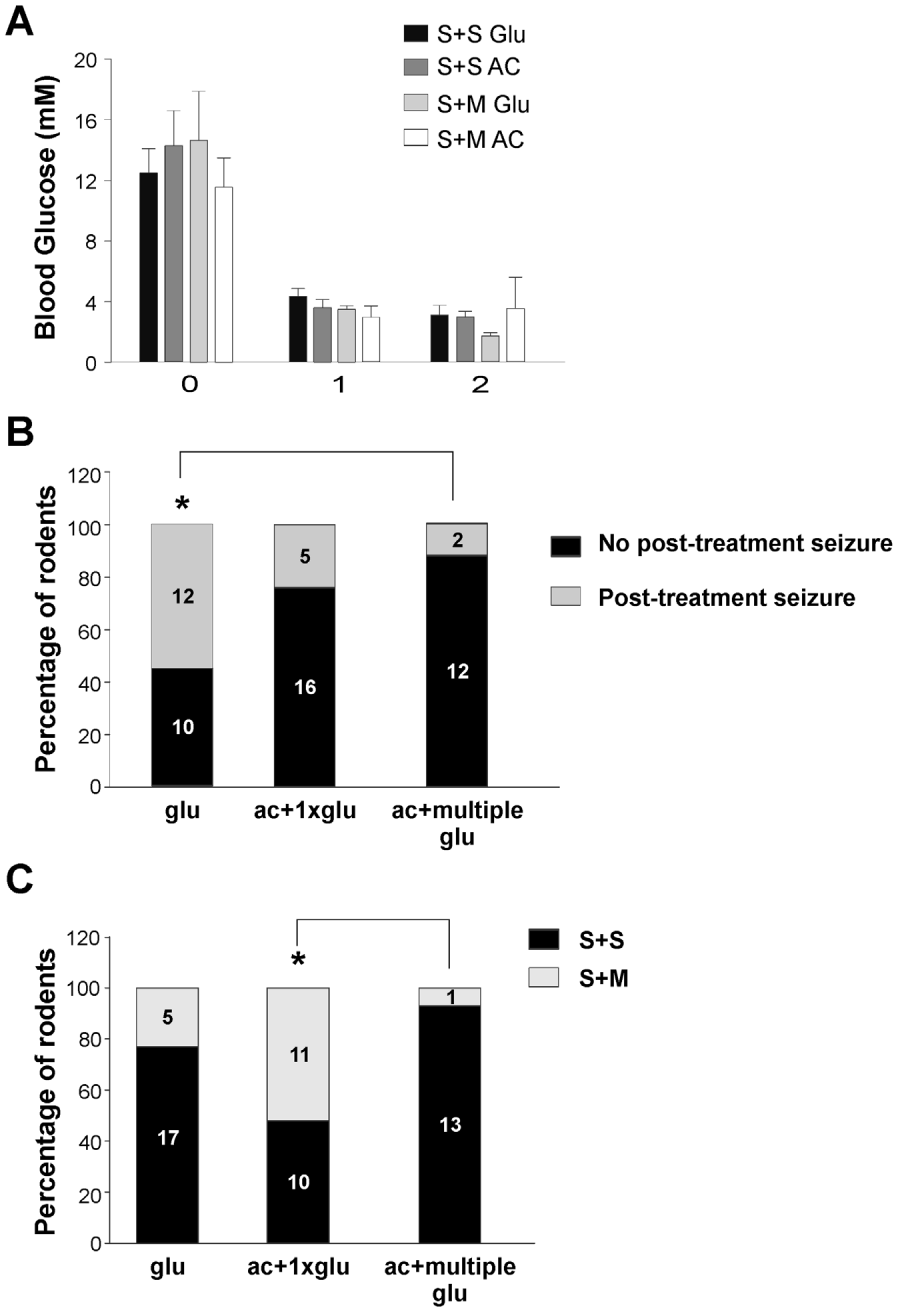
Efficacy of treatment strategies (see Table 1) in preventing subsequent seizures and mortality in STZ rats. **A**: BG decrease is not significantly different in seizing animals regardless of treatment; **glu**: seizure+ survival (S+S Glu), **ac+1xglu**: S+S AC, **glu**: seizure + mortality (S+M Glu), **ac+1xglu**: S+M AC (Table 4). B: No significant difference in the incidence of seizures post-treatment in **ac+1xglu**: 24% (n = 5/21) compared with glu: 55% (n = 12/22) (p<0.05). (*) Significantly lower incidence of seizures post-treatment in **ac+multiple glu**: 15% (n = 2/14) compared with **glu**: 55% (n = 12/22) (p<0.02) C: (*) Significantly higher survival rate in **ac+multiple glu**: 93% (n = 1/14) compared with **ac+1xglu**: 48% (n = 10/21) (p<0.02). No significant difference in survival rate between **ac+multiple glu**: 93% (n = 13/14) and **glu**: 77% (n = 17/22).

To evaluate the efficacy of glucose, the current treatment strategy, and whether outcomes differed with anticonvulsant treatment, the treatments described in **Table 1** (**glu, ac+1xglu, ac+multiple glu)** were administered to STZ rats. The trend in BG decrease after insulin was similar in the treatment groups, indicating that the BG decline was not a confounding factor of treatment outcome (**Figure 5A** **)**.

**Table 4:** Mean blood glucose level measured hourly related to treatment and survival.

	N	0 hr	N	1 hr	N	2hr
S+S AC+1XGLU	10	14.3±2.3	9	3.6±0.5	4	3.0±0.4
S+S Glu	17	12.5±1.6	17	4.3±0.5	13	3.2±0.6
S+M AC+1XGLU	11	11.6±1.9	5	3.0±0.7	3	3.5±2.1
S+M Glu	5	14.7±3.2	5	3.5±0.2	3	1.7±0.2

S+S Glu: Seizure + Survival; glucose treated.

S+S AC+1XGLU: Seizure + Survival; glucose and anticonvulsant treated.

S+M Glu: Seizure + Mortality; glucose treated.

S+M AC+1XGLU: Seizure + Mortality; glucose and anticonvulsant treated.

Treatment with glucose at seizure onset was not always successful in mitigating seizures. 55% (n = 12/22) of **glu** treated animals exhibited seizures after treatment compared with 24% of animals in the **ac+1xglu** treatment group (n = 5/21) (**Figure 5B**). In the **ac+multiple glu** group, repeated glucose administration at BG<2.5 mM, significantly reduced the incidence of post-treatment seizures compared to the **glu** group with only 15% of rodents (n = 2/14) undergoing subsequent seizures (p<0.02) (**Figure 5B**). There was not a significant difference between the **ac+1xglu** (n = 5/21) and the **ac+multiple glu** (n = 2/14).

Despite the success of anticonvulsant treatment in mitigating seizures with multiple glucose administrations, the impact on survival, **ac+multiple glu**: 93% (n = 13/14), compared with **glu**: 77% (n = 17/22) was not significant (**Figure 5C**). However, the survival rate of **ac+multiple glu**: 93% was significantly higher than **ac+1xglu:** 48% (n = 11/21; p<0.02). As the anticonvulsants appeared to be mitigating the motor component of the seizures (**Figure 5B** **)**, despite continued hypoglycemia (**Figure S3 A-B**), improved survival rate was still dependent on continued glucose administration.

### Anticonvulsants suppresses motor seizures

**Figure 6:**
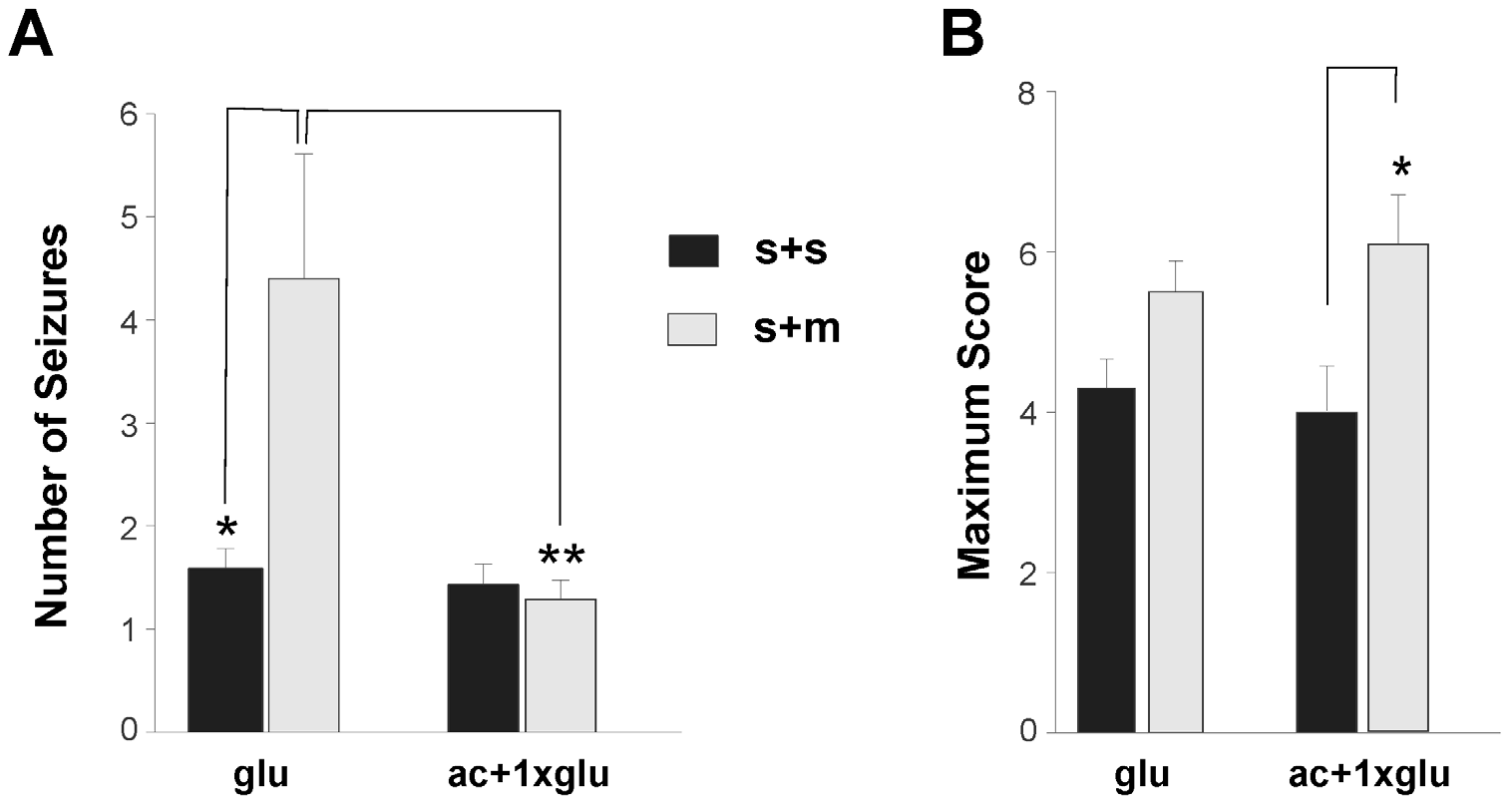
Effects of treatments (see Table 1) on the seizure scores and mortality in STZ rats. **A:** Mean number of seizures in (*) **glu** rats: S+S: 1.6±0.2 (n = 17) and S+M: 4.4±1.2 (n = 5) (p<0.001); **ac+1xglu** rats: S+S: 1.3±0.2 (n = 8) and S+M: 1.4±0.2 (n = 8). (**) Significant difference also exists between **glu** rats: S+M and **ac+1xglu** rats: S+M (p<0.05) **B:** Mean maximum seizure score attained in **glu** rats: S+S: 4.3±0.4 (n = 17) and S+M: 5.5±0.4 (n = 5); (*) **ac+1xglu** rats: S+S: 4.0±0.6 (n = 8) and S+M: 6.1±0.6 (n = 8) (p<0.01).

As demonstrated in **Figure 5B**, combining diazepam and phenytoin with glucose was more successful at mitigating convulsive behaviour than glucose alone. In the **ac+1xglu**-treated group the number of motor seizures were similar whether or not mortality resulted (S+S: 1.3±0.2; n = 8 and S+M: 1.4±0.2; n = 8) and significantly lower (p<0.05) than the **glu**-treated rats where mortality resulted (S+M: 4.4±1.2; n = 5) (**Figure 6A** **)**. In **ac+1xglu** group, the 8 animals in the S+M group died despite having a reduced number of seizures, as glucose was necessary even though the anticonvulsants prevented the behavioural seizure manifestations. However, multiple glucose administrations (**glu**) alone were not sufficient to prevent continued seizures that eventually led to mortality (**Figure 6A** **)**. These rats may have had a temporary increase in **BG** levels and returned to hypoglycemia. In the **ac+multiple glu** group only one animal died and could not be statistically compared to the other 2 groups (S+M: 3.0; n = 1).

Interestingly, the maximum seizure score in the **ac+1xglu**-treated group was significantly higher in S+M rats: 6.1±0.6 (n = 8) compared to S+S rats: 4.0±0.6 (n = 8) (p<0.01). While anticonvulsants were able to transiently stop seizing, since the rats were not treated with continuous glucose, 5 rats eventually had more severe seizures. In contrast, the maximum score observed in the **glu**-treated rats that survived (4.3±0.4; n = 17) was statistically similar to those that died (5.5±0.4; n = 5) (**Figure 6B** **)**. This was also the case in **ac+multiple glu**-treated rats: S+S: 4.0±0.4 (n = 13) and S+M: 5.5 (n = 1), though the sample size of the latter was not adequate for statistical analysis.

### EEG seizure activity is not associated with motor seizures

**Figure 7:**
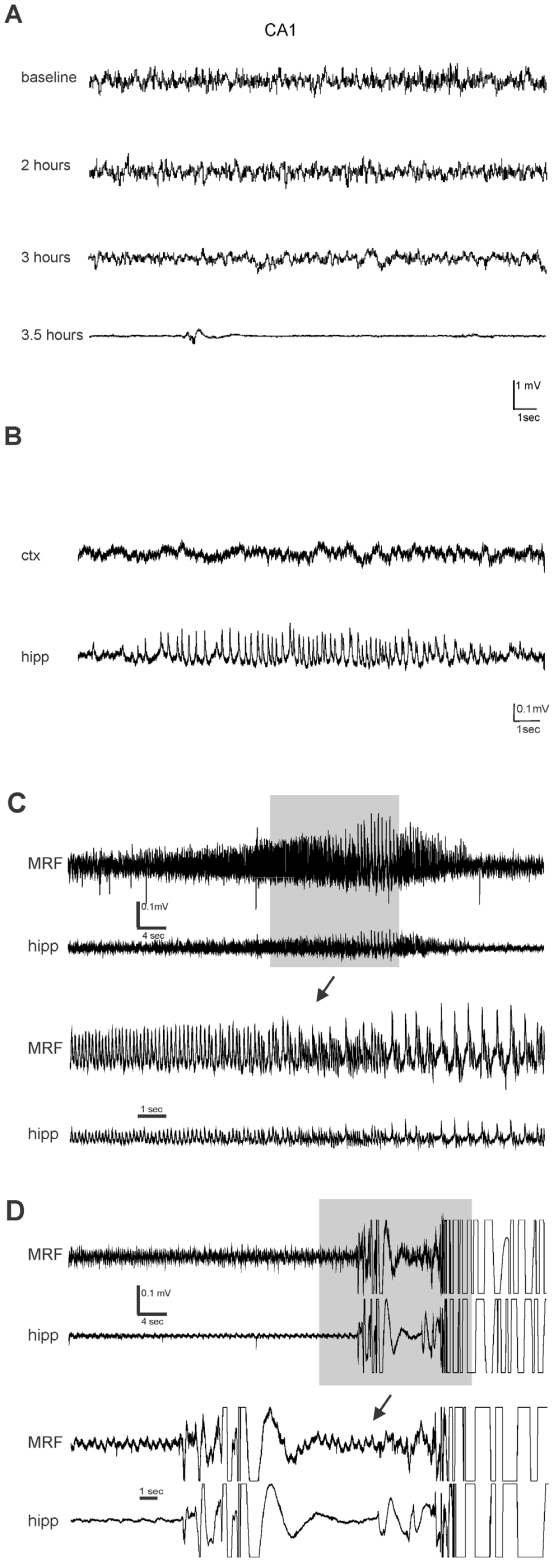
EEG abnormalities during hypoglycemia is not associated with seizure behavior. **A:** Representative EEG recording of hippocampus (CA1) at baseline (post-fasting; prior to insulin IP), 2 hours, 3 hours (slower waves) and 3.5 (EEG suppression) hours after insulin IP **B:** Electrographic seizure activity observed in CA1 (lower trace) after suppression of EEG activity. No ictal activity in cortex (upper trace) **C:** EEG recording of hippocampus and contralateral MRF obtained in STZ rat during hypoglycemia. Electrographic seizure activity observed after suppression of EEG activity. Lower trace illustrates magnification of the area in the gray box **D:** EEG of the rat in (**C**) during a behavioural seizure; ictal activity may be masked by movement artifact.

EEG recordings were obtained in 17 STZ and 12 CON rats. 11/12 CON and 13/17 STZ animals reached hypoglycemic levels following insulin administration of which 10 CON and 9 STZ rats had behavioral seizures. An example of baseline recording prior to insulin administration is shown in **Figure 7A**. 3 of the 13 hypoglycemic STZ rats showed significant suppression (**Figure 7A** **)** of EEG, characteristic of severe hypoglycemia (Auer 2004). However, behavioural seizures occurred independently of EEG suppression as only 2 out of 9 STZ and none of the CON rodents displayed such pronounced suppression prior to exhibiting convulsions. Burst suppression was characteristic of EEGs in all animals that reached hypoglycemic levels. Animals that seized showed intermittent, non-rhythmic spikes, often with a positive polarity. Two animals’ recordings also contained low-amplitude (50 uV) polyspike bursts. Brief electrographic seizures were recorded in the CA1 (**Figure 7B** **)** region in one animal and brainstem (**Figure 7C** **)** in another. Although, rats at this stage of hypoglycemia were lethargic and immobile, no overt convulsive behaviour was observed. The rat exhibiting the hippocampal electrographic seizure had previously received a “rescue” administration of glucose, diazepam and phenytoin after the prior incidence of a behavioural seizure. The other had not been treated as a behavioural seizure had not yet been observed.

Consistent with previous studies [41], the behavioural manifestations classified as “seizures” were not associated with any characteristic recordings. **Figure 7D** illustrates motion artifact induced by seizure-like behavior, but no rhythmic or paroxysmal change in the EEG immediately before, or during the seizure-like behaviour.

### Hypoglycemic seizures are associated with hippocampal and cortical damage

**Figure 8:**
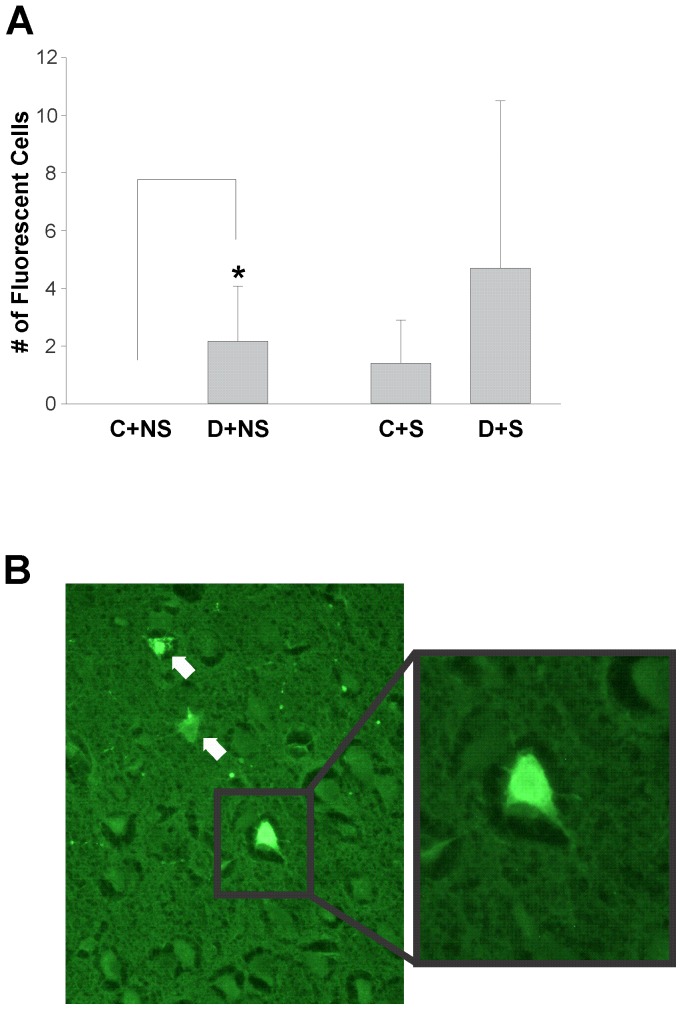
Effects of diabetes and seizures on neuronal damage. **A:** Significant difference in the number of fluorojade (+) cells between CON non-seizing (C+NS: 0±0; n = 5 animals) and STZ non-seizing groups (D+NS; 2.17±1.9 cells; n = 6; p<0.05). No significant difference in the number of cells between CON seizing (C+S: 1.4±1.5; n = 5 cells) and STZ seizing groups (D+S: 4.7±5.8 cells; n = 7) **B:** Fluorojade (+) cells in the cortical region of (D+S) rat magnified 40X (white arrows).

Fluorojade staining was performed to visualize degenerating cells and assess the effects of hypoglycemic seizures on the neurons of STZ and CON animals. The number of fluorescent cells was minimal in the four groups: CON and STZ rats dissected 48 hours after insulin-induced hypoglycemic seizures and age-matched non-seizing CON and STZ animals. STZ rats without seizures (D+NS: 2.17±1.9 cells; n = 6) exhibited a significantly higher number of damaged cells than CON rats (C+NS: 0±0; n = 5 cells; p<0.05). There was no significant difference between CON (C+S: 1.4±1.5 cells; n = 5) and STZ rats (D+S: n = 7; 4.7±5.8 cells) particularly due to the high variability in the number of damaged cells in the STZ animals. Two STZ rats displayed numerous (>10) fluorojade (+) cells while this others exhibited more minimal damage (**Figure 8**). Consistent with previous literature, neurodegenaration was localized to the cortical and hippocampal regions [16], [26].

## Discussion

This study has established a model of hypoglycemic seizures in young diabetic animals to examine seizure manifestations and consequences in this age group. As Type 1 diabetes is diagnosed primarily during childhood, these hypoglycemic episodes begin early in life and the associated neurological complications are particularly harmful [5], [13], [48]. Repeated hypoglycemic seizures in young children may also cause structural brain changes. Impaired awareness is particularly prevalent when the age of diabetes diagnosis is early (<6 years) and is associated with recurrent hypoglycemic episodes resulting in seizures or coma [48] though other studies have reported conflicting results [18], [20]. In addition, animal studies have demonstrated the increased susceptibility of the juvenile brain to neuronal excitability and seizures [34], [36], [49] and shorter latency to seizure generalization [45]. While there are several clinical studies that have assessed the effects of seizures such as EEG abnormalities [37] and increased cognitive impairmanet [50], the specific incidence of hypoglycemic seizures is unknown.

In our model, the drop in blood glucose (BG) within the first hour post-insulin administration was predictive of those animals that would attain severe hypoglycemia and progress to seizures (**Figure 2A** **/B)**. In STZ animals, the higher baseline fasting BG levels resulted in lower incidence of hypoglycemia and seizures (**Figure 1A** **/B)**. These higher initial BG levels in STZ rats combined with the similar rates of BG decline following insulin administration resulted in an increased latency to hypoglycemia and subsequent seizures in these animals.

Blood glucose measured at seizure onset provided evidence that there is a limited range of BG levels at which seizures occur and is an indication of a glycemic threshold for seizures. Despite expectations that the STZ animals would have increased seizure susceptibility [31], seizure-threshold BG levels were similar between the STZ and CON groups. A potential reason for this similarity is the brief period of diabetes in this study. Streptozotocin could only be administered in post-weaned (21-day old) animals and hypoglycemia was induced after a week in 28–29 day old rats. This age was chosen, as it is neuro-developmentally similar to young children, a population at risk for hypoglycemic episodes [51]. Additionally, Veliskova et al [45] reported the decreased latency to tonic-clonic seizures, associated with brainstem involvement, in this age group. As such, this age was maintained in our experiments to avoid additional variability in the results of the study. Recurrent hypoglycemic episodes occur more often in diabetic patients and therefore the resulting impaired counterregulation [9] may lower seizure threshold. These experiments were the first step in creating a model of hypoglycemic seizures in juvenile diabetic animals; not yet reported in the literature to our knowledge. Future experiments will evaluate the impact of recurrent hypoglycemic seizures in diabetic juvenile/younger mature animals chronically treated with insulin, as this is more representative of the clinical situation.

Mortality was only observed in animals post-seizure. Additionally, the animals that survived, despite having similar seizure scores, underwent a significantly lower number of seizures (CON: S+S  =  1.6±0.3; n = 11, S+M  =  7.8±2.7; n = 5 p<0.01 and STZ rats: S+S  =  1.6±0.2; n = 17, S+M  =  4.4±1.2; n = 5 p<0.001; **Figure 4C** **).** This was further evidence for the association between seizures and mortality. Velisek et al, [32] showed that persistently low BG, despite multiple glucose administrations, was the potential cause of continued seizures. BG measured post-seizure demonstrated variable rates of recovery from seizures with some animals recovering only temporarily and returning to hypoglycemic levels (Figure S4). Further evidence for impaired recovery is that a single glucose dose was insufficient to ameliorate seizures in 12/22 rats. Four rats continued seizing despite multiple glucose treatments with mortality as an end result.

The behavioural presentation of seizures in this model suggests brainstem involvement due to the loss the righting reflex and rolling seizure manifestations [45]. Furthermore, mortality was always observed in rats that attained a seizure score of 7 (this score reflects full tonic extensions), suggesting brainstem involvement in the seizures where mortality resulted. Disruption in the functioning of brainstem regions may cause cardiac and respiratory changes that can lead to mortality [52]. Even animals that did not die naturally but had to be sacrificed (2 CON and 7 STZ rats; Table S1) due to their severe state displayed severe gasping/agonal breathing.

The survival rates were significantly higher in the **ac+multiple glu** compared with the **ac+1xglu** group. The correction of hypoglycemia with multiple administrations of glucose, where necessary, was beneficial for survival (**Figure 5A**). Anticonvulsant treatment at seizure onset coupled with multiple glucose administrations significantly reduced subsequent convulsions compared with the **glu** treated group (**Figure 5B**) with no significant effect on mortality. Interestingly, the BG levels remained lower, despite repeated glucose administrations (Figure S3 B), with decreased seizure occurrence potentially due to the presence of the anticonvulsants.

Diazepam, a GABA agonist, and phenytoin, which act on sodium channels, were only able to transiently stop seizures without glucose replenishment and the seizures eventually become more severe with mortality as an end result (**Figure 6B** **).** This temporary effect suggests that other seizure-causing mechanisms, such as enhanced NMDA activity, may trigger these seizures in the presence of continued hypoglycemia [42], [53]. This emphasizes the importance of repeated glucose replenishment to prevent both seizure severity and mortality. Even though anticonvulsants decreased the behavioural manifestations of seizures the rats may have continued to seize non-convulsively, particularly in the group only receiving a single dose of glucose treatment, as the energy substrate was not continuously replenished. This can have clinical implications as anticonvulsants may be administered to prevent continued seizures that exacerbate the neuronal damage caused by hypoglycemia [26], but it remains essential to replenish the energy substrate.

Consistent with previous literature, neuronal degeneration was negligible as BG levels were not maintained at the very severe hypoglycemic level of ≤ 1.0 mM with suppressed EEG, as was done experimentally by Auer [54]. Our results demonstrate that seizures occur at higher BG levels, ∼ 2.0 mM. Notably, there were significantly more fluorojade positive cells in non-seizing diabetic animals compared with non-seizing controls. Hyperglycemia can exacerbate neural damage caused by other pathologies such as seizures and ischemia [26], [55]–[57]. Yet, hyperglycemia per se has not been shown to cause neurodegeneration in adult animals [26]. Furthermore, Sasaki-Hamada et al [44] demonstrated that rats incurring diabetes at a juvenile age exhibited increased cognitive deficits compared to adult animals. Our data provide further evidence that studies on hypoglycemic seizures should be performed in a young diabetic model due to the differences in the outcome between adult and juvenile animals. However, due to the high variability of damage in seizing STZ rats, there was no significant difference between CON and STZ animals that seized.

As observed in previous studies in adult rodents [41], our EEG data provide further evidence that behavioral seizures in juvenile diabetic animals do not originate in the hippocampus. There were no apparent predictive EEG changes in the STZ and CON rats. Severe hypoglycemia (≤1.0 mM) as indicated by EEG isoelectricity [24] was not a requirement for convulsions; 18 of 19 rodents had behavioral seizures without pronounced suppression of EEG. There was slowing in the EEG waves prior to seizures in these animals, indicative of less severe hypoglycemia (>1.0 mM) [54]. It is not surprising that EEG isoelectricity is not associated with seizures since the BG of ≤1.0 mM, a BG level that is rarely reached by patients [58], was not a requirement for seizures. This was confirmed in **Figure 3A** where the mean BG at seizure onset was 1.6±0.1 mM in CON and 1.8±0.2 mM in STZ rats.

Electrographic seizures recorded in the hippocampus (+/–brainstem) were brief (10–30s), failed to spread to the cortex, and occurred in the context of EEG suppression that is associated with neuronal damage [54]. These observations suggest that the same mechanisms responsible for neurotoxicity may be crucial in sustaining the depolarization of susceptible neuronal networks, but the lack of adequate energy substrate could prevent spread to higher brain regions. In addition, the observed seizure-like behavior (some of which could be interpreted as primitive locomotion phenomenology) as seen in decerebrate preparations [59] suggests a brainstem or spinal cord generator. Further studies recording EEG activity from other brainstem and spinal regions are required to test this hypothesis.

There are several factors that potentially explain the mechanisms for seizures during hypoglycemia. The downregulation of K_ATP_ channels that impair potassium buffering has been postulated to enhance excitability [32]. The decrease in intracellular ATP impairs the activity of the Na^+^/K^+^-ATPase and thus the cell’s ability to restore intracellular potassium and extracellular sodium levels, leading to an enhanced glutamate receptor function [60]. This is further exacerbated by the increase in excitatory amino acids such as glutamate and aspartate as well as ammonia [54], [61] that has been correlated with the onset of seizures [25]. This increase in production of glutamate and aspartate is the result of accumulating oxaloacetate from an impaired Kreb’s cycle [54]. Additionally, increase in extracellular glutamate can saturate glutamate receptors in astrocytes as well as cause astrocytic swelling particularly in the cortex and hippocampus [62]. The ability of AP7, a glutamate receptor antagonist, to significantly mitigate hypoglycemic motor seizures is evidence for the crucial role of excitatory amino acids in these convulsions [42]. The increased excitability and possibly decreased inhibition can cause synchrony of neuronal firing, which can be propagated by electrical conduction through gap junctions [63]–[65].

In summary, hypoglycemic seizures due to exogenous insulin administration can occur in juvenile diabetic and non-diabetic animals. A blood glucose threshold of approximately 2mM was associated with the occurrence of seizures in both control and diabetic animals after an acute hypoglycemic event. Most importantly, mortality only occurred following seizures, and it was the frequency of seizures rather than blood glucose levels that were predictive of mortality. Treatment of seizures with glucose, helped prevent seizure-related mortality with no added protection from anticonvulsants. While electrographic seizures were recorded in two animals, it was not correlated with behavioural manifestations. Although there have been improvements in treatments such as better insulin, insulin pumps, and improved glucose monitoring techniques, hypoglycemic seizures remain a major problem in insulin-treated diabetic children and adolescents, and a better understanding of these is crucial for improved patient care. The description of the seizures and outcomes performed in our STZ-model is the first step toward accomplishing this goal.

## Supporting Information

Figure S1 **Blood Glucose (BG) measured post seizure of glu-treated CON and STZ animals. A:** BG post-seizure in CON rats at 1.0 hour (n = 8): 4.4±0.2 mM, 2.0 hours (n = 8): 5.5±0.5 mM **B:** BG post-seizure in STZ rats 1.0 hour (n = 11): 7.8±1.9 mM, 2.0 hours (n = 12): 13.7±2.9 mM, 3.5 hours (n = 5): 15.9.3±4.1 mM.
(TIF)

Figure S2 **Evolution of seizures over the course of hypoglycemia (each trace represents a different animal). A:** CON; S+S; n = 11 **B:** CON; S+M; n = 5 **C:** STZ; S+S; n = 17 **D:** STZ; S+M; n = 5.
(TIF)

Figure S3 **Blood Glucose (BG) post seizure of glu and ac+multiple glu-treated STZ animals. A:** BG post-seizure in **glu** rats 1.0 hour (n = 11): 7.8±1.9 mM, 2.0 hours (n = 12): 13.7±2.9 mM, 3.5 hours (n = 5): 15.9.3±4.1 mM **B:** BG post-seizure in **ac+multiple glu** rats at 0.5 hours (n = 12): 3.1±0.5 mM, 1.5 hours (n = 10): 3.2±0.4 mM, 2.5 hours (n = 6): 3.2±0.6 mM, 3.5 hours (n = 4): 6.3±3.3 mM.
(TIF)

Figure S4 **Total duration in which the rodents demonstrated SLEs as an indication of hypoglycemia.** No significant difference between glucose-treated control rats that seized and died; CON_GLU_S+M (n = 5): 124.3±31.5 and survived; CON_GLU_S+S (n = 11): 42.1±20.1 No significant difference between glucose-treated diabetic rats that seized and died; STZ_GLU_S+M (n = 5): 113.6±38.0 and survived; STZ_GLU_S+S (n = 17): 55.8±16.2 No significant difference between anticonvulsants+1 dose of glucose-treated diabetic rats that seized and died; STZ_ACGLU1_S+M (n = 8): 30.7±11.4, and survived; STZ_ACGLU1_S+S (n = 8): 36.1±12.0 Anticonvulsants+multiple glucose-treated diabetic rats that seized and survived; STZ_ACMULTGLU_S+S (n = 13): 97.04±26.2 could not be statistically compared with ones that seized and died; STZ_ACMULTGLU_S+M (n = 1).
(TIF)

Table S1 **Reasons for sacrificing 2 of 5 glu Treated CON rats, 2 of 5 glu treated STZ rats and 5 of 8 ac+1xglu treated STZ rats.**
(DOC)
