# Supplementary figures and images for: Severe Hypoglycemia in a Juvenile Diabetic Rat Model: Presence and Severity of Seizures Are Associated with Mortality
Source: PLoS One. 2013 Dec 30;8(12):e83168. doi: 10.1371/journal.pone.0083168 (PMC3875447; doi:10.1371/journal.pone.0083168)

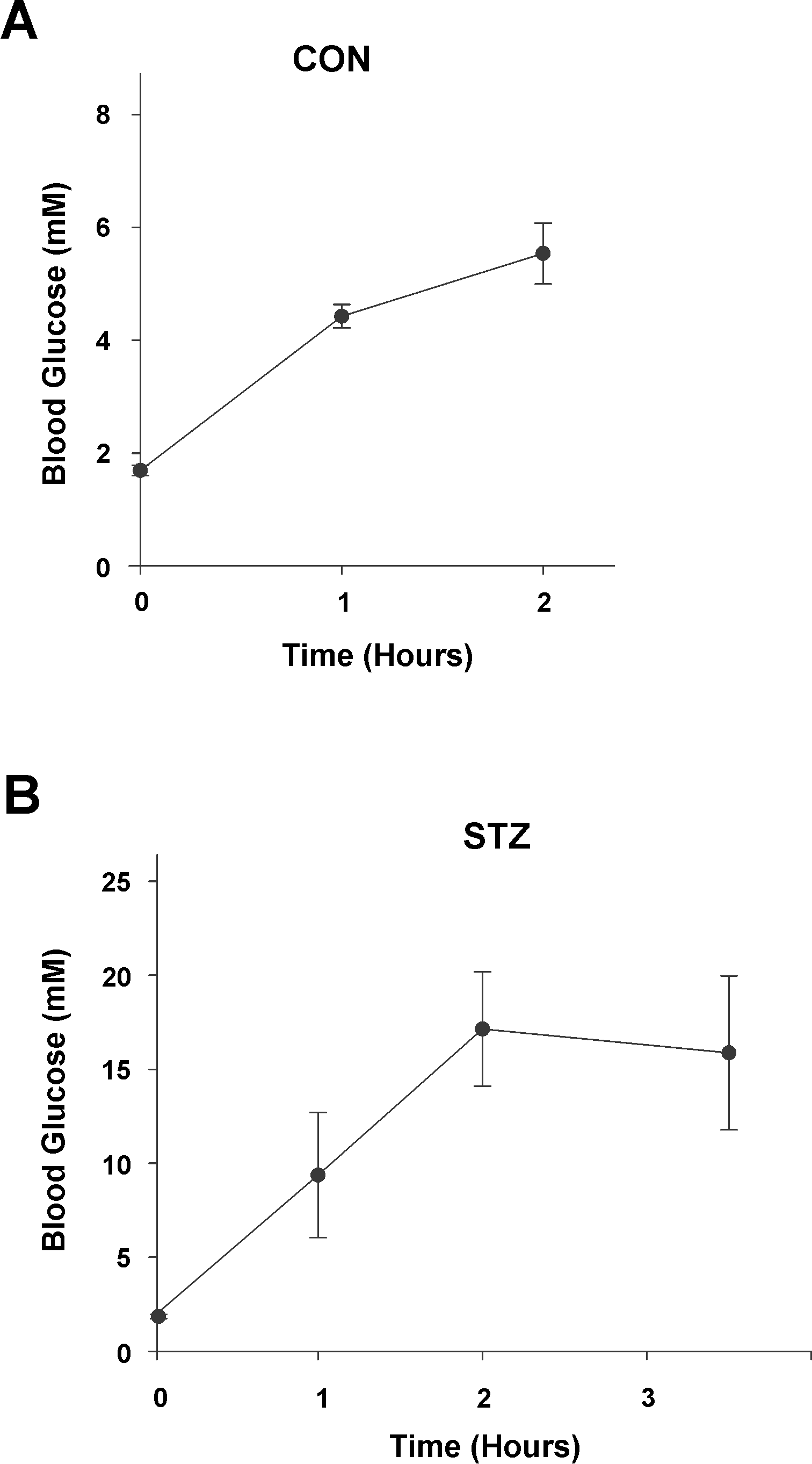

Supplement: Figure S1 — Blood Glucose (BG) measured post seizure of glu-treated CON and STZ animals. A: BG post-seizure in CON rats at 1.0 hour (n = 8): 4.4±0.2 mM, 2.0 hours (n = 8): 5.5±0.5 mM B: BG post-seizure in STZ rats 1.0 hour (n = 11): 7.8±1.9 mM, 2.0 hours (n = 12): 13.7±2.9 mM, 3.5 hours (n = 5): 15.9.3±4.1 mM. (TIF) [file pone.0083168.s001.tif]

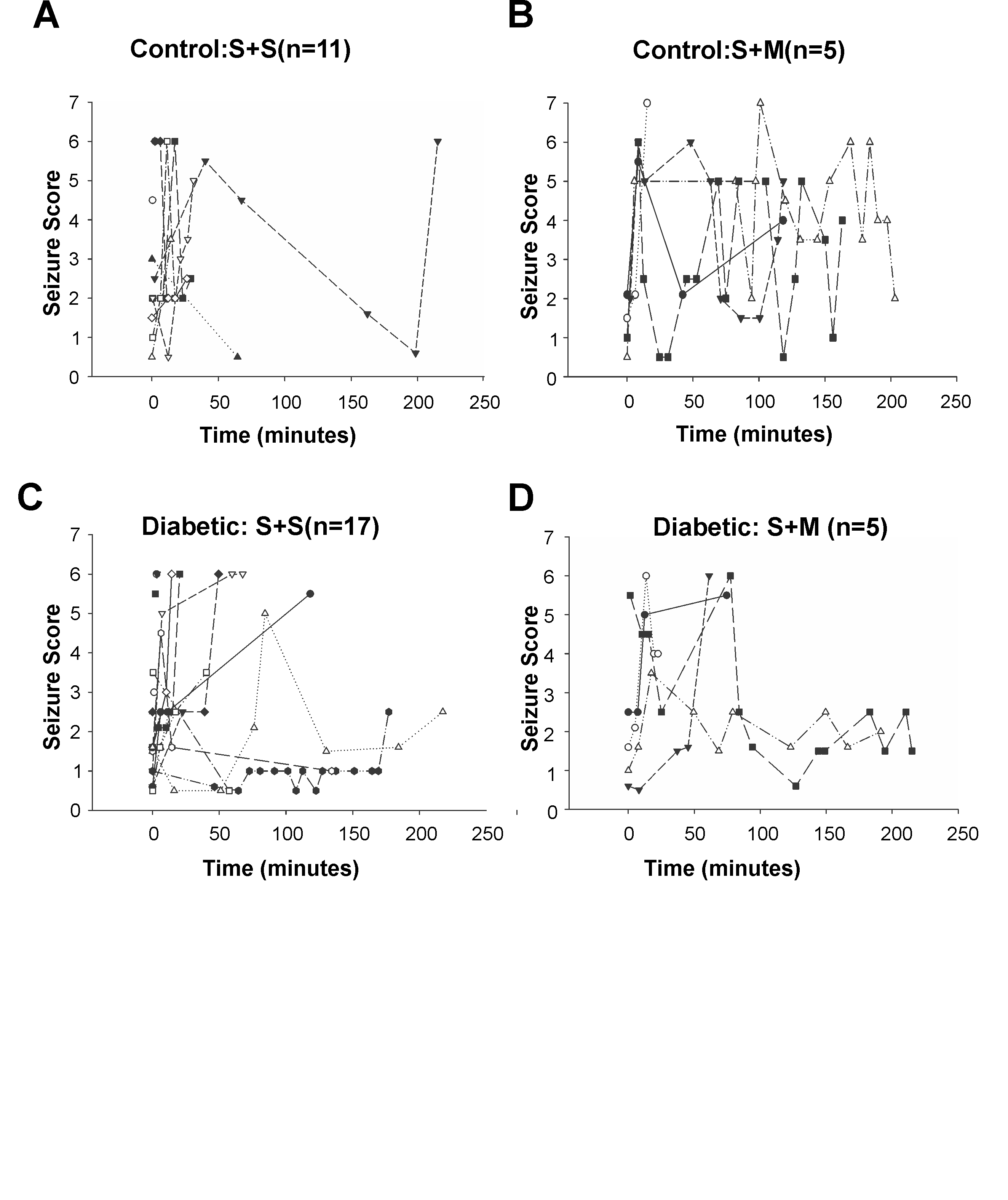

Supplement: Figure S2 — Evolution of seizures over the course of hypoglycemia (each trace represents a different animal). A: CON; S+S; n = 11 B: CON; S+M; n = 5 C: STZ; S+S; n = 17 D: STZ; S+M; n = 5. (TIF) [file pone.0083168.s002.tif]

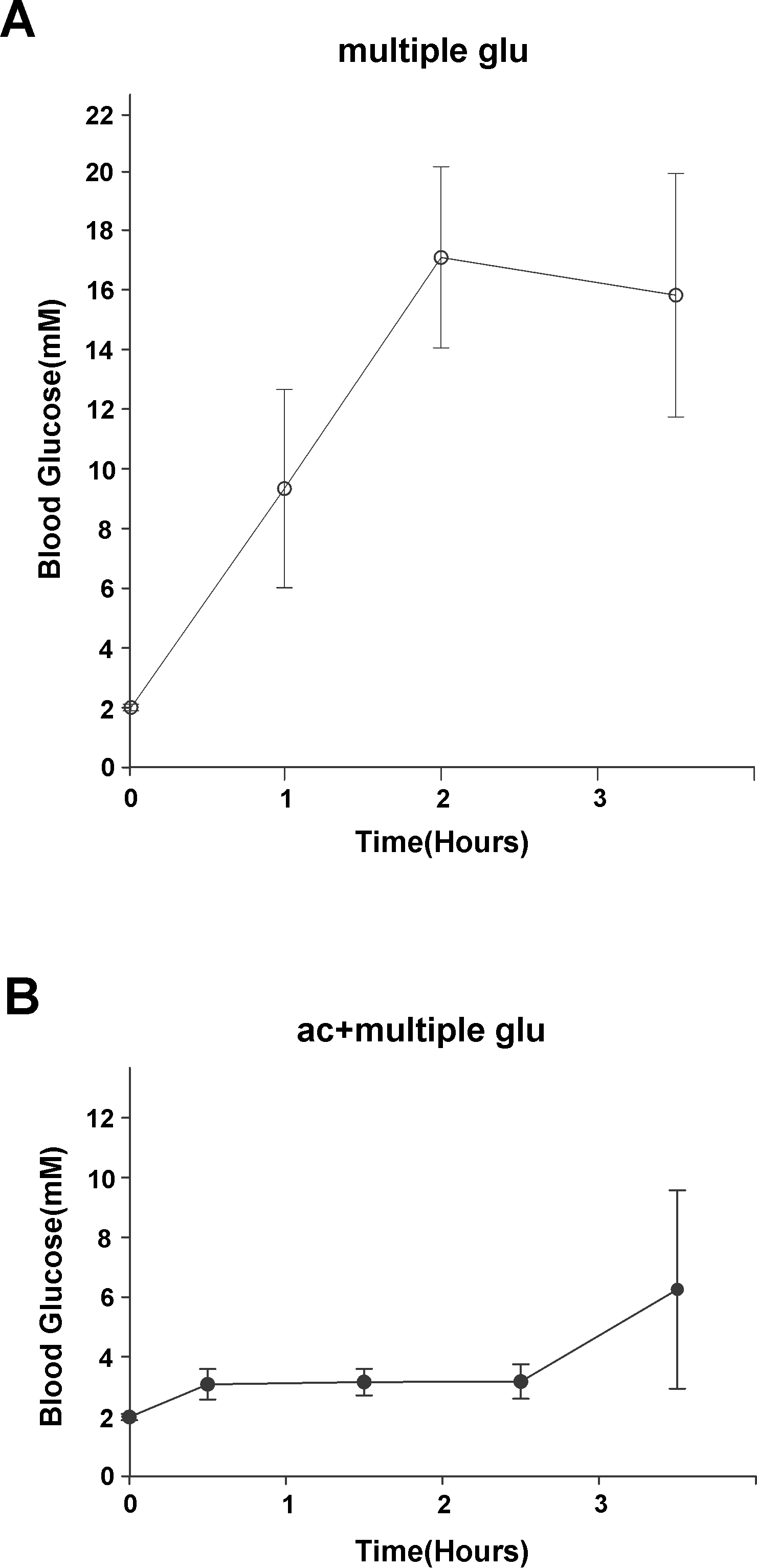

Supplement: Figure S3 — Blood Glucose (BG) post seizure of glu and ac+multiple glu-treated STZ animals. A: BG post-seizure in glu rats 1.0 hour (n = 11): 7.8±1.9 mM, 2.0 hours (n = 12): 13.7±2.9 mM, 3.5 hours (n = 5): 15.9.3±4.1 mM B: BG post-seizure in ac+multiple glu rats at 0.5 hours (n = 12): 3.1±0.5 mM, 1.5 hours (n = 10): 3.2±0.4 mM, 2.5 hours (n = 6): 3.2±0.6 mM, 3.5 hours (n = 4): 6.3±3.3 mM. (TIF) [file pone.0083168.s003.tif]

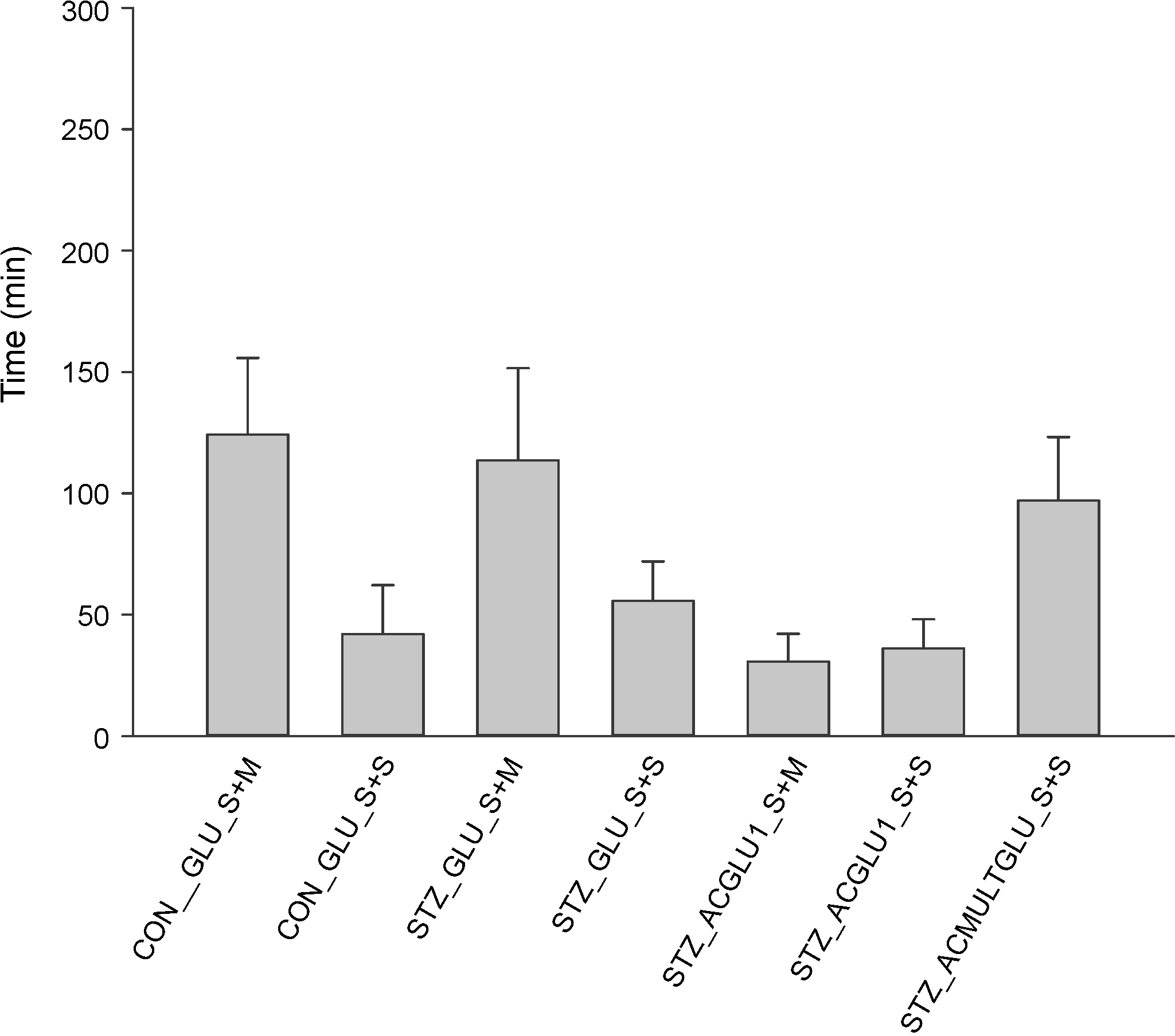

Supplement: Figure S4 — Total duration in which the rodents demonstrated SLEs as an indication of hypoglycemia. No significant difference between glucose-treated control rats that seized and died; CON_GLU_S+M (n = 5): 124.3±31.5 and survived; CON_GLU_S+S (n = 11): 42.1±20.1 No significant difference between glucose-treated diabetic rats that seized and died; STZ_GLU_S+M (n = 5): 113.6±38.0 and survived; STZ_GLU_S+S (n = 17): 55.8±16.2 No significant difference between anticonvulsants+1 dose of glucose-treated diabetic rats that seized and died; STZ_ACGLU1_S+M (n = 8): 30.7±11.4, and survived; STZ_ACGLU1_S+S (n = 8): 36.1±12.0 Anticonvulsants+multiple glucose-treated diabetic rats that seized and survived; STZ_ACMULTGLU_S+S (n = 13): 97.04±26.2 could not be statistically compared with ones that seized and died; STZ_ACMULTGLU_S+M (n = 1). (TIF) [file pone.0083168.s004.tif]
