# Supplementary material for: Severe Hypoglycemia in a Juvenile Diabetic Rat Model: Presence and Severity of Seizures Are Associated with Mortality
Source: PLoS One. 2013 Dec 30;8(12):e83168. doi: 10.1371/journal.pone.0083168 (PMC3875447; doi:10.1371/journal.pone.0083168)
Supplement: Table S1 — Reasons for sacrificing 2 of 5 glu Treated CON rats, 2 of 5 glu treated STZ rats and 5 of 8 ac+1xglu treated STZ rats. (DOC) [file pone.0083168.s005.doc]

**Table S1**

| **Rat #** | **Type** | **Treatment** | **Time Unresponsive (min)** | **# SLEs** | **Reason for Sacrifice** |
| --- | --- | --- | --- | --- | --- |
| 7-22-11-6 | CON | GLU | 120 | 6 | Immobile and unresponsive between seizures |
| 7-22-11-8 | CON | GLU | 135 | 11 | Moved head once between seizures but did not show normal behavior; i.e. eating and grooming |
| 1-19-11-10 | STZ | GLU |  | 3 | Continued seizing at the end; the final seizure was unresponsive to treatment |
| 7-13-11-1 | STZ | GLU | 85 | 8 | Multiple seizures not responsive to repeated glucose administrations |
| 6-11-10-16 | STZ | AC+1XGLU | 102 | 1 | Moved head once 112 mins after treatment but was otherwise immobile and did not eat |
| 6-11-10-15 | STZ | AC+1XGLU | 39 | 1 | Began gasping 40 mins after treatment |
| 6-18-10-14 | STZ | AC+1XGLU | 22 | 1 | Tonic extensions 15 minutes after treatment and began gasping for breath |
| 6-30-10-5 | STZ | AC+1XGLU | 21 | 1 | Tonic extensions 21 mins after treatment and feet turned blue |
| 6-30-10-6 | STZ | AC+1XGLU | 15 | 2 | Tonic extension 15 mins after first seizure and several gasps for breath |
